# What have Vietnamese scholars learned from researching entrepreneurship? A Systematic review

**DOI:** 10.1016/j.heliyon.2020.e03808

**Published:** 2020-04-24

**Authors:** Quan-Hoang Vuong, Viet-Phuong La, Thu-Trang Vuong, Hong-Kong T. Nguyen, Manh-Tung Ho, Manh-Toan Ho

**Affiliations:** a Centre for Interdisciplinary Social Research, Phenikaa University, Hanoi 100803, Viet Nam; b A.I. for Social Data Lab (AISDL), Vuong & Associates, Dong Da district, Hanoi 100000, Viet Nam; c Sciences Po Paris, Campus de Dijon, 21000 Dijon, France; d Institute of Philosophy, Vietnam Academy of Social Sciences, Ba Dinh district, Hanoi, 10000, Viet Nam

**Keywords:** Entrepreneurship, Entrepreneurship research output, Creativity, Innovation, Economics, Economic development, Business, Management

## Abstract

This study reviews the research landscape of entrepreneurship studies done by Vietnamese researchers from 2008 to 2018. A sample size of 111 articles from 108 academic outlets (journals, conferences proceedings, and book chapters) indexed in Web-of-Science and Scopus were extracted on the SSHPA database, then read and systematically classified into 15 topics. A systematic review reveals (i) a high frequency of research on various aspects of management, (ii) a lackluster focus on innovation and creativity in entrepreneurial activities, (iii) and worrisome cultural influences on the level of creativity. Overall, there was evidence of a detachment between the academic community and the entrepreneurial community. The research landscape shows there have not been enough studies done on the following aspects of entrepreneurship: technology application, poverty reduction, network development, internationalization, inter-generational transfer, and sex/gender.

## 1 Introduction

### 1.1 A Western-centric field

The extant scholarship on entrepreneurship was shown to be dominated by studies coming from advanced industrialized English-speaking countries such as the United States, the United Kingdom, and Canada (Meyer et al., 2014). Bibliometric studies on entrepreneurship have investigated how the field has emerged and transformed over time (Meyer et al., 2014), or the field's research front (Cornelius et al., 2006), or the process of specialization and the rise of networks (Schildt et al., 2006). The scholars have recognized it is necessary to comprehend the role of entrepreneurship in developing countries in finer details (Acs et al., 2008; Autio, 2008; Naudé, 2009; Thurik and Audretsch, 2001). Nonetheless, there have only been country-specific bibliometric studies on major emerging markets such as China and India (Sharma, 2019; Zhai et al., 2014). Smaller economies have largely been overlooked. Since examining entrepreneurial activities without its institutional, socio-economic, and cultural context would present a barrier for a comprehensive understanding, this study aims to contribute to the literature by finding out patterns in the research landscape of studies on entrepreneurship in Vietnam, a growing lower-middle-income country, through its own object-oriented structured dataset. The next section seeks to explain why Vietnam is a suitable context for this study.

### 1.2 Vietnam: Economic transformation and entrepreneurial activities

From a command economy under the poverty line, after on three decades of reforms, Vietnam's *per capita* income grew two-fold from USD202 in 1986 to USD417 in 2001, then jumped to over USD2,500 then by 2018 (Pham and Vuong, 2009; Vuong, 2019a). This transformation can also be seen in the number of operating firms, from 1991 to 1999, around 40,000 new firms were established, and this number rose steeply by nearly 18-fold to over 700,000 from 2000 to 2018 (Vuong, 2019a). With the dominance of small and medium enterprises (SMEs), entrepreneurship can be viewed as the backbone of the economy (Pham and Vuong, 2009; Vuong, 2019a; Vuong and Tran, 2009). There were just about 37,700 SMEs in the 2000s, while in 2015, there was more than 413,000, an equivalent of 93.5% of the total. The average registered capital per firm also increased two-fold to over VND53 billion (USD2.43 million) during that period (Vietnam General Statistics Office, 2017).

In recent years, the wave of entrepreneurial activities became ever stronger, and the country has become known for its increasing favorable conditions for entrepreneurship. In 2018, according to the London-based Global Entrepreneurship Research Association (2018), Vietnam was among those with the highest total early-stage entrepreneurial activity—23.3%—and a significant entrepreneurial spirit index—0.26. Given entrepreneurship's important historical role in Vietnam's growth story, and that it is such an essential point in all current policy discussions, it is important to establish the landscape of research in the recent decade. This study performs a systematic review of 111 studies conducted by Vietnamese researchers and published in Web-of-Science/Scopus-indexed journals from 2008 to 2018. As such, the current study is among the first attempts to review the research output and the research landscape regarding entrepreneurship in this fast-growing economy. This study will highlight the general research trends over the past 10 years and under-researched topics within entrepreneurship studies in Vietnam. In doing so, we hope not only to provide valuable insights for future research direction but also to generate high-quality information for policy-makers to make more informed macro-economic decisions.

## 2 Materials and method

This section presents a description of our database system and data collection procedure in full details. Further details can be found in Vuong et al. (2020).

### 2.1 SSHPA data analysis or SDA

The review was enabled by SSHPA (Social Sciences & Humanities Peer Award), which is an open database built for the purpose of tracking publications in Web-of-Science/Scopus-indexed academic outlets (including journals, conference proceedings, and book chapters) of Vietnamese researchers in social sciences and humanities. The starting point of data collection is 2008, and it is an on-going national project whose data quality has been validated through a series of publications attempting to find correlates of productivity of Vietnamese researchers. Among the publications, the most notable is the data descriptor paper or the method paper reviewed by Nature Research's Scientific Data, which demonstrates the full potential for reproducibility of the data collecting and cleaning procedure (Vuong, La, Vuong, Ho, et al., 2018a). SDA is short for SSHPA Data analysis. SDA can extract data from SSHPA and produce a wide range of data visualizations. These two steps of data processing allow the resulting datasets to be structured, organized, accurate, and customizable for specific purposes, such as a literature review of a particular discipline.

### 2.2 Search strategy and classification of topics

First, the study performs a search within the SSHPA database using the following search keywords: entrepreneurship; entrepreneur; entrepreneurial firms and their synonyms such as small and medium enterprises; small business; startup; micro firms and microfinance. The result is a total of 111 research articles from 108 academic outlets published in the period from 2008 to 2018. All articles’ abstracts were then reviewed by the research team to ensure each article matches the inclusion and exclusion criteria. Admittedly, the search keywords can be widened, which might result in a large sample size. However, given that this study is country-specific, and limited to the period from 2008 and 2018, the number of articles in the sample is comparable to other review studies done in a much larger scale. For example, Kiss et al. (2012) reviewed 156 articles from 14 journals on international entrepreneurship in emerging economies; Müller (2016) reviewed 170 articles from 10 journals on regional entrepreneurship; Terjesen et al. (2013) reviewed 259 articles from 21 journals international entrepreneurship from 1989 to 2010; Sutter et al. (2019) reviewed 200 articles from 77 journals on entrepreneurship and poverty alleviation from 1990 to 2017. All of the review articles cited above state that their search strategy only focuses on leading/premier academic journals regarding entrepreneurship. Arguably, this strategy might introduce certain biases into the sample size; for example, research from developing countries where academic research community are still in the nascent phase might not be included. Moreover, this search strategy might not be applicable to an emerging economy/academic community such as Vietnam, where research output is still relatively low. Consequently, our methodology, to an extent, brings about a novelty to the current literature.

After double-checking the 111 articles, the authors classified the articles based on proposed a list of eight key topics: Corporate social responsibility; Business efficiency; Innovation and creativity; Gender/sex; Organizational management; Resources Management; and Inter-generational transition. For an article to belong to any one of the above topics, the topic must be analyzed and discussed thoroughly in the articles’ result and discussion section. For example, when a topic is only briefly mentioned then, the article would not be counted toward the topic. Next, the proposed list of topics was refined by two independent researchers who read the full text of the articles and examined any newly proposed topics. Group discussions among research team members are conducted to settle all disputes on inclusion or exclusion of topics. Finally, seven more topics are added: Legal and institutional matters; Internationalization, Entrepreneurial education; Poverty alleviation and Job creation; Network development; Motivations and Values; Technological utilization.

Using a similar procedure, this study identifies five research methods: interview, questionnaire, case study, literature review and theoretical research (non-data approach). The attributes of the articles are then coded into the SDA system to generate data visualizations to further check for errors. The dataset is publicly available on OSF (DOI: 10.17605/OSF.IO/NJMSY / URL: https://osf.io/njmsy/) (Ho et al., 2019). After that, a systematic content review is conducted involving a large group of researchers. This process takes advantage of the synergy among computational algorithms, structured datasets, and human researchers; thus, it is hoped to minimize the biases and errors when dealing with a large number of data.

## 3 What do entrepreneurship researchers actually write about?

### 3.1 Research output trend regarding topics

In terms of the 15 topics as categorized by this review paper, the management group (see Table 1) contains *Organizational management* (discussed in 59 articles)*, Resources management* (53)*, Legal and institutional matters* (39), *Business efficiency* (38). Clearly, studies over the past decade have built on early scholarship which showed human resources, capital, and government's support were the challenges for Vietnamese entrepreneurs (Ardrey et al., 2006; Leshkowich, 2006; Ronnas, 2001; Truong, 2002).

**Table 1:** Finalized list of entrepreneurship research topics.

No.	Topics	Number of Articles	Examples of Studies
1	Social and cultural influences	36	(Dang et al., 2016; Nguyen and Nordman, 2018; Nguyen and Mort, 2016; Nguyen and Rose, 2009; Perri and Chu, 2012; Vuong, 2016a; Vuong and Napier, 2015)
2	Resources management	53	(Chu and Luke, 2012; Duy and Oanh, 2015; Nguyen et al., 2015; O'Cass et al., 2012; Pham and Talavera, 2018; Turkina & Thi Thanh Thai, 2013; Thai and Ho, 2010; Vuong, Do and Vuong, 2016a)
3	Business efficiency	38	(Hiep and Van Vu, 2015; Hoa and Khoi, 2017; Lensink and Pham, 2012; Nguyen et al., 2013; Santarelli and Tran, 2013; Tran and Santarelli, 2017; Walder and Nguyen, 2008)
4	Innovation and creativity	38	(Brundenius and Le, 2014; Kiura et al., 2014; Le et al., 2018a; Nam et al., 2017; Tuan, 2015; Vuong and Napier, 2015; Vuong et al., 2013)
5	Gender/Sex	8	(Le et al., 2016; Nguyen, Frederick and Nguyen, 2014a; Oosterhoff and Hoang, 2018; Poon et al., 2012; Smith et al., 2017)
6	Inter-generational transition	14	(Le et al., 2018a; Ngo et al., 2018; Pham et al., 2018; Tuan, 2015; Thu Hien and Santarelli, 2012; Trong Tuan, 2017a)
7	Management	59	(Carbonara et al., 2018; Cox and Le, 2014; Dung and Janssen, 2015; Le, 2015; Nguyen, 2015; Nguyen et al., 2016; Nguyen and Pham, 2017; O'Cass and Ngo, 2011; Suzuki et al., 2014; Tran et al., 2016; Vuong, 2016c; Vuong, Vu and Vuong, 2016b)
8	Corporate social responsibility	28	(Luu, 2017; Napier and Quan Hoang, 2011; Nguyen and Nordman, 2018; Quan and Huy, 2014; Thai and Chong, 2013; Thi Bich, Huu Chi, Thi Xuan Mai, & Thi Phuong Thao, 2012; Vuong, 2016b; Walder and Nguyen, 2008)
9	Legal and institutional matters	39	(Carbonara et al., 2016; Dang, 2011; Hoa and Khoi, 2017; Nguyen and Le, 2019; Nguyen and Mort, 2016; Nguyen and Nguyen, 2011; Nguyen, 2014; Thu Hien, 2018; Tran et al., 2016; Vu and Le, 2016)
10	Internationalization	17	(Nguyen et al., 2008; Nguyen et al., 2013; Thai et al., 2012; Vu and Lim, 2013; Vu et al., 2016)
11	Education and Training	10	(Ngo et al., 2018; Pham et al., 2018; Raven and Le, 2015; Suzuki et al., 2014; Vuong, La, Vuong, Nguyen, et al., 2018b; Vuong and Napier, 2015)
12	Poverty alleviation and job creation	21	(Cox and Le, 2014; Noel et al., 2010; Nguyen, Verreynne and Steen, 2014b; Paswan and Tran, 2012)
13	Network Development	17	(De Jong, Tu and van Ees, 2012; Nguyen, 2014; Nguyen et al., 2016; Vu, Napier and Vuong, 2013b)
14	Values and motivations	22	(Mai and Chong, 2008; Nguyen and Nguyen, 2008; Turkina & Thi Thanh Thai, 2015; Vuong and Napier, 2014)
15	Technological utilization	3	(Hoang and Swierczek, 2008; Le et al., 2018b; Le et al., 2012).

The next group examines and outlines the typical features of entrepreneurial behavior in Vietnam, namely *Innovation and creativity* (38), *Corporate social responsibility* (38), *Social and cultural influences* (36), and *Motivations and values of entrepreneurs* (22). The period from 2012 onwards witnessed a surge of studies on Innovation and creativity, which has remained one of the most investigated topics in this group. There was a noticeably high number of articles concerning social and cultural issues in 2016, followed by a sharp decrease in the following year – this observation is explained by the overall drop of articles in the field and suggests that the rise was only temporary.

Lastly, the emerging topics concern *Poverty alleviation and Job creation* (21), *Internationalization* (17), *Network development* (17), *Inter-generational transition* (14), *Education and training* (10), *Gender/sex* (8), *Technological application* (3). These topics were often only discussed alongside with the main and foundation topics. In general, the number of articles in this group remains low and not consistent. An observable trend here is the slight boost of articles addressing the issue of Network development in 2016, which mirrors the patterns of research about Law, regulation and institutions. This could signify the connections among topics within the field since the issue of Network development often involves discussions about corruption and bribery with implications for institutions and policy.

### 3.2 Strong focus on management

As noted in Table 1 and the section above, the research landscape in Vietnam on entrepreneurship skewed toward various aspects of entrepreneurial management rather than cognitive and theoretical aspects of entrepreneurship, which are the dominant subject areas in the world (Ferreira et al., 2019b; Meyer et al., 2014). The most consistent and outstanding finding is the heavy concern of firms for managing financial capital (Thu Hien and Santarelli, 2014; Tran, Abbott and Yap, 2017; Vu et al., 2016; Vuong, Do, et al., 2016a). In order to optimize firms’ capability to pursue different growth objectives including better access to credit, a common suggestion is entrepreneurial firms should use their networks of business partners according to different stages of development (Le and Nguyen, 2009; Nguyen and Nguyen, 2011; Pham and Talavera, 2018; Thai and Ho, 2010; Vu, Napier and Vuong, 2013a).

Another aspect of management can be seen among the articles within the *Legal and Institutional matters* topic. Legal and institutional challenges for private firms in Vietnam require not only the government to step up but also proactive actions from entrepreneurs. Studies find that policies are inconsistent in nature, prone to changes, and sometimes target at only a small group of SMEs (Nguyen and Pham, 2017). The consensus is: even though Vietnam has shifted from centrally planned to market economy, a higher degree of liberalization and transparency of its legal frameworks are still required to provide a more favorable free-market competition that encourages the growth of private sector enterprises (Carbonara et al., 2018; Le et al., 2012; Nguyen and Pham, 2017; Paswan and Tran, 2012; Pham and Talavera, 2018; Turkina & Thi Thanh Thai, 2013; Thai and Chong, 2013).

The complex and unpredictable legal practices in a transitional context trap business in Vietnam into a hidden web of trust-based connections (Napier and Vuong, 2013). Driven by a set of unwritten rules, businesses end up paying out of their own pockets to smoothen operations, thereby raising entrepreneurial transaction costs. The act of “giving an envelope” in Vietnam, however, carries a deeper meaning: it is about establishing a mutually beneficial relationship between the entrepreneurs and the officials involved. In this sense, the institutional barriers temporarily break down, leaving room for personal maneuvers. In the literature, some findings have suggested bribery or “speed money” can surprisingly help firms reduce costs arising from the lack of security and transparency (Tran et al., 2016; Vu, Tran, Nguyen and Lim, 2018). However, corruption does have a negative impact on firms' productivity and their compliance with state regulations (Tran et al., 2016; Vu and Le, 2016; Vu et al., 2018). In transitional economies such as Vietnam, the rampant corruption is often rooted in the institutional structure, which results in the government's discrimination between the state-owned and private sectors (Nguyen and Rose, 2009). Researchers reported a lack of measures and support programs targeted at sector-specific organizations (Cox and Le, 2014; T. Pham and Talavera, 2018). Given that existing policies are not yet protective of the rights and needs of firms (Hoa and Khoi, 2017; Thai and Ho, 2010), more attention should be given to better allocate national resources among state and non-state sectors to eradicate any discrimination and promote efficiency and productivity of both sectors (Nguyen and Mort, 2016; Thi Nhung, Gan and Hu, 2015). Specific suggestions include reducing tax and improving loan access (Hoa and Khoi, 2017), decentralizing policy-making to better support SMEs (Cox and Le, 2014; Le et al., 2016; Paswan and Tran, 2012; Tran and Santarelli, 2017), and increasing trust in the government at the community level (Dut, 2015; Nguyen and Wongsurawat, 2012; Phuong, 2014).

### 3.3 Lackluster focus on creativity and innovation

**Figure 1:**
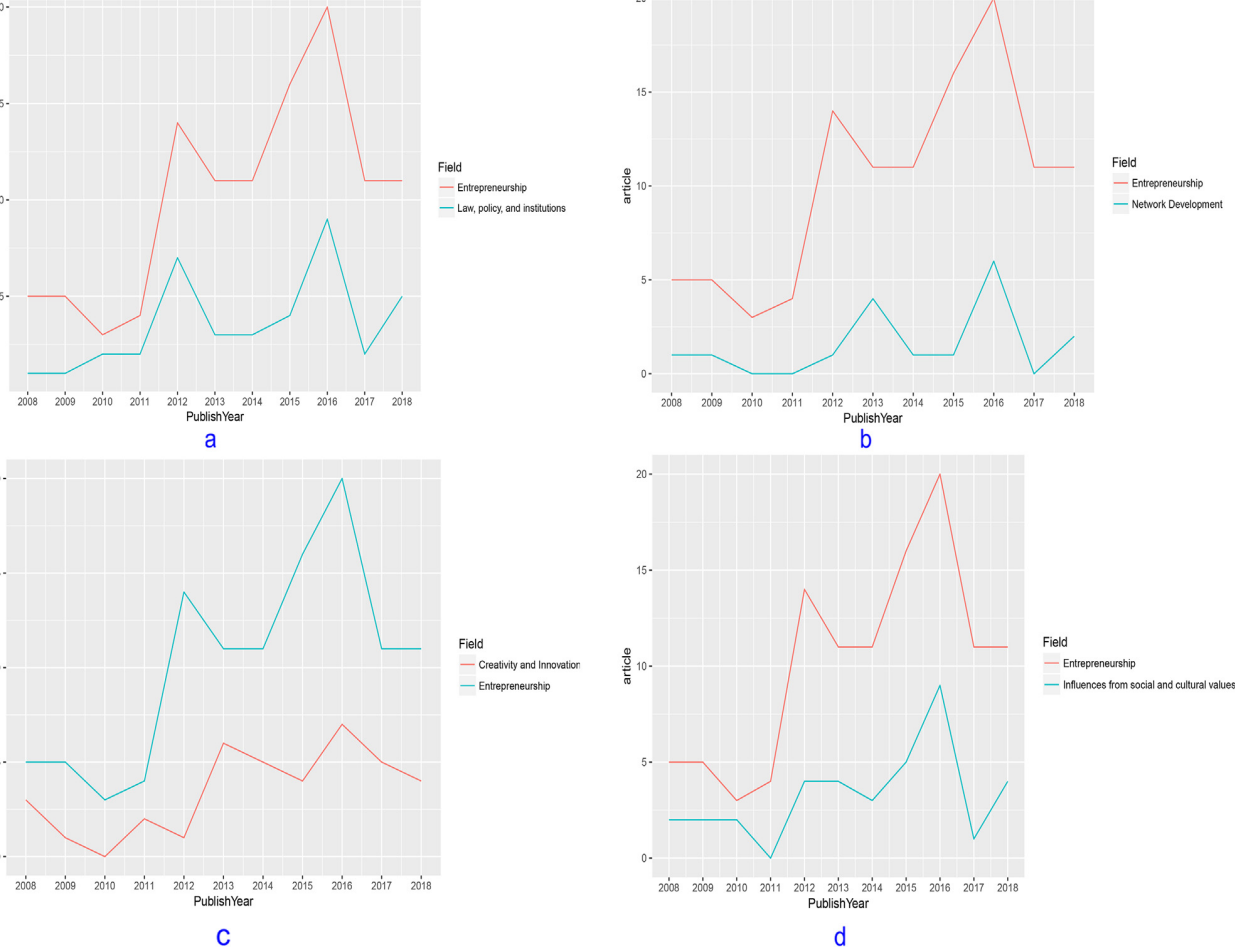
The trends in entrepreneurship research on a) Legal and institutional matters, b) Network development, c) Innovation and Creativity, and d) Social and cultural influences.

With a significant proportion of research dedicated to innovation and creativity, several findings are important and worthy of attention not only from the academic community but also the policymakers and businesses. Innovation and creativity are perceived to be costly and only applicable for top-level managers (Brundenius and Le, 2014; Le, Dao, Pham and Tran, 2019; Nguyen and Nguyen, 2011; Nguyen and Pham, 2017; O'Cass and Ngo, 2011). In terms of remedies, researchers have repeatedly suggested all levels of an entrepreneurial organization to promote new initiatives so as to be more proactive to changes in a fast-moving market (Luu, 2017; Nam et al., 2017; Tuan, 2015, 2017; Thai and Chong, 2013; Vu et al., 2013a). This also means that managers should motivate member staff to accommodate new values and be more open to necessary changes, be it organizational structure, working process or potential market (O'Cass et al., 2012). Leaders should also exert moderate tolerance of failures and mistakes to encourage an innovative culture in the company (Le, 2015; Trong Tuan, 2017b). A large dataset of 759 responses from managers and employees (Trong Tuan, 2017a) suggests that collaboration and knowledge sharing should be stimulated within an organization as the initial step towards innovation (see Figure 1).

### 3.4 Cultural influences on entrepreneurship

**Figure 2:**
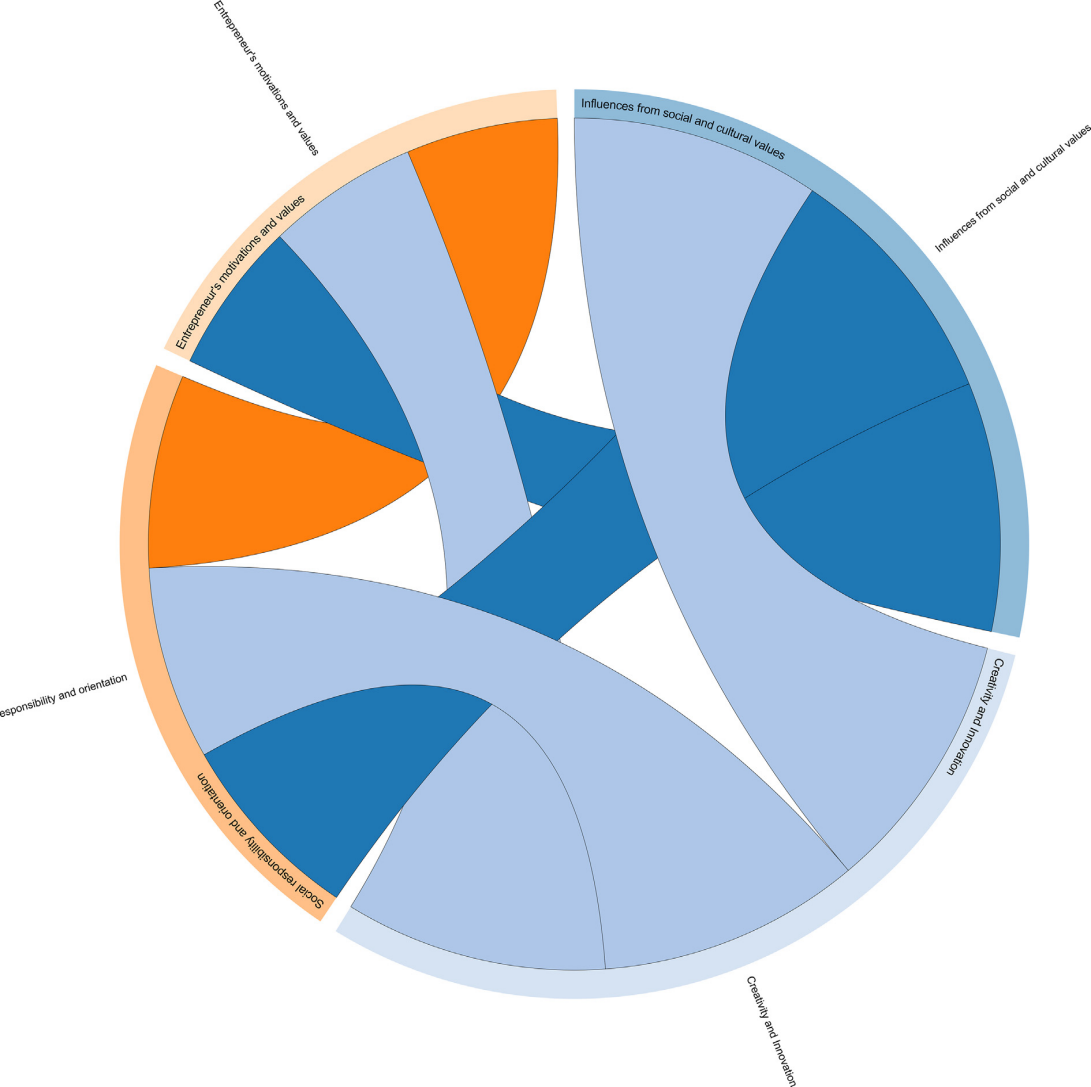
Chord diagram shows connections among Creativity and innovation, Influences from social and cultural values, and Motivations and values of entrepreneurs topics.

Yet, it is important to note that the cultural factor may lie behind why much of the non-innovative working culture in enterprises across Vietnam has persisted so long. As pointed out in a number of previous studies, elements of a predominant Confucian culture can downplay the value of contribution as well as status of entrepreneurs (Nguyen, Bryant, Rose, Tseng and Kapasuwan, 2009; Thai and Nguyen, 2016; Vuong, La, Vuong, Nguyen, et al., 2018b). Indeed, casting against the background of a collectivist culture like Vietnam, entrepreneurial goals such as personal development, pursuit of passion or adventure seeking can be seen as individualistic. Those are, in fact, not as a strong motivation as the desire to provide jobs for family members (Perri and Chu, 2012; Pham et al., 2018). Here, our data analysis allows a visualization of the connection among some sub-topics in research entrepreneurship, shedding light on the often-obscure influences from social and cultural values (Figure 2).

Even more notably, Confucian philosophy has been found to associate with Vietnamese limited innovation capacity, tolerance of uncertainty, and submissiveness to environmental changes (Nguyen and Rose, 2009). It is common for entrepreneurs to start businesses by initially copying the model from other existing firms and amend them later in order to adjust to the changing environment. Findings have also revealed that the majority of business founders in Vietnam base their initial settings on personal intuition and pure luck without any analytical plan carried out (Nguyen and Nguyen, 2008; Nguyen et al., 2009; Poon et al., 2012). With regards to operating the business, in-depth interviews with 12 entrepreneurs show that luck continues to be significant in finding opportunities from a limited personal network while entrepreneurs do not actively search for opportunities or sources of information (Thai and Nguyen, 2016). It should nonetheless be noted that studies did highlight different findings among regions within the country. The effects of traditional values tend to be perpetuated in northern areas where these features are more deeply rooted (Le et al., 2016). The findings overall raise doubts on whether Vietnamese entrepreneurial ventures are truly creative endeavors or representative of the additivity of blind venturing efforts.

### 3.5 Under-researched topics

**Figure 3:**
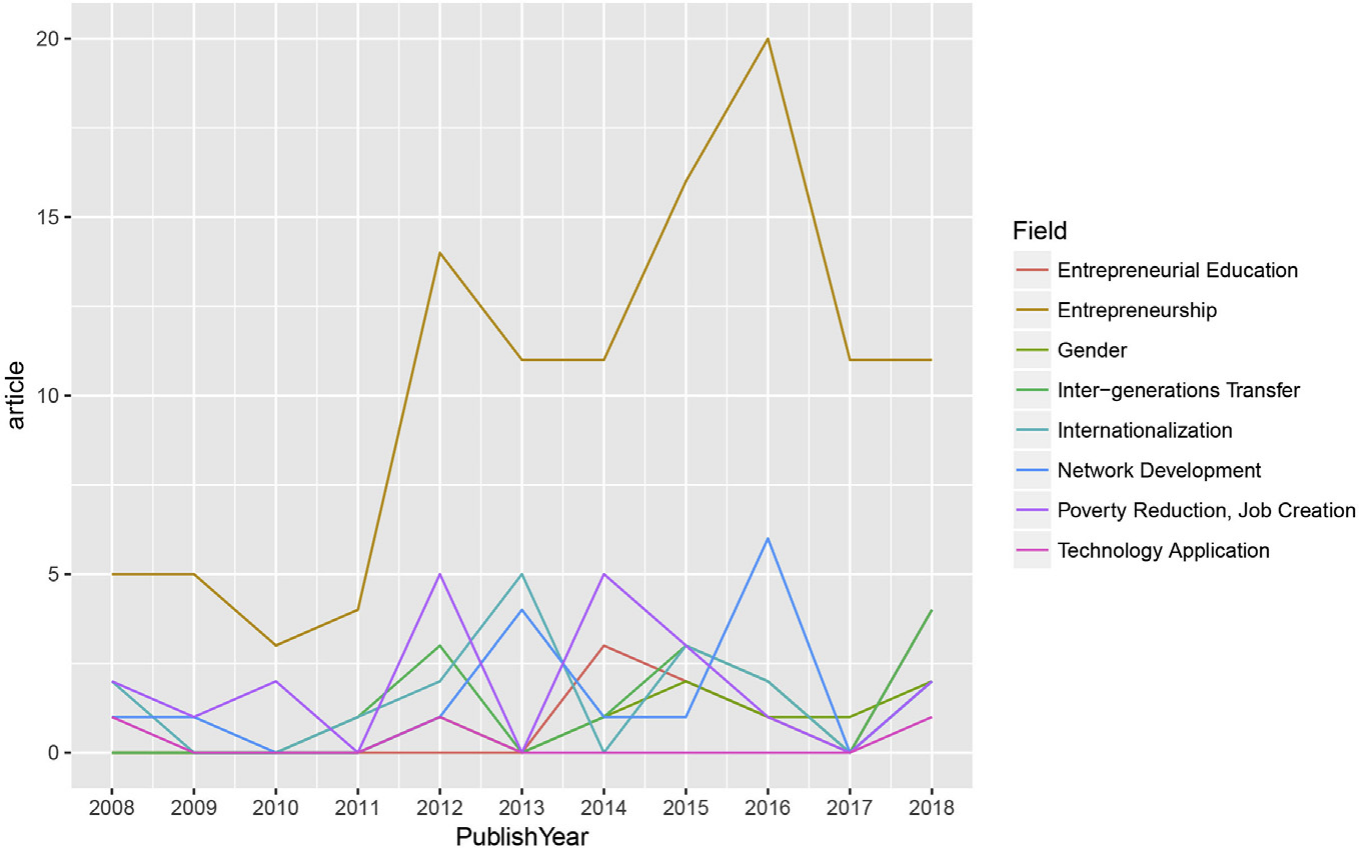
The emerging topics in Entrepreneurship in Vietnam, 2008–2018.

Besides the topics that received more coverage in the 2008–2018 period, there are some notable topics that paled in comparison. Figure 3 shows the lack of attention for entrepreneurship research with a focus on technology application, poverty reduction, network development, internationalization, inter-generational transfer, and gender.

It is indeed striking that, although *Industry 4.0* and *Artificial Intelligence* have become the buzzwords in both governmental agendas and public discussions in recent years (VNS, 2018), technological application is nearly absent from much of the research on entrepreneurship in Vietnam. In reality, many Vietnamese entrepreneurial mobile applications have successfully exploited the opportunity such as Foody, GoViet, or Rada. However, from 2008 to 2018, there have only been 3 research articles that can be classified into the topic of *Technological Utilization* (Hoang and Swierczek, 2008; Le et al., 2018b; Le et al., 2012). None of those studies mention high-tech applications such as machine learning, artificial intelligence, etc.

Equally disheartening is the topic of gender/sex in entrepreneurship, which only has eight articles from 2008 to 2018. A consistent finding is women, especially who are in the rural areas, and the transgender population stand to gain from taking entrepreneurial initiatives (Le et al., 2016; Nguyen, Frederick, et al., 2014a; Poon et al., 2012; Raven and Le, 2015; Smith et al., 2017, yet holding them back are issues such as societal judgement, capital constraints, and limited education are still crippling them (Nguyen et al., 2014a; Oosterhoff and Hoang, 2018; Raven and Le, 2015). These results might become obsolete as according to a recent survey, 25% of Vietnamese women, out of necessity, participate in entrepreneurial activities, marking a higher proportion than men (22%) (VCCI, 2018). Once again, it can be argued that academic research is lagging behind the evolution of entrepreneurship in this aspect.

## 4 Discussion and conclusion

In sum, this study has reviewed the literature of entrepreneurship research in Vietnam from 2008 to 2018, bringing attention to the (i) high frequency of research on various aspects of management of enterprises, (ii) lackluster focus on innovation and creativity in entrepreneurial activities, (iii) and worrisome cultural influences on the level of creativity. More importantly, the low level of research output and the existence of several under-researched topics display a lack of engagement between scholar and entrepreneur communities. The wedge between the entrepreneurs and the researchers is reflected most clearly in the low number of entrepreneurship research that tackles technology adaptation and gender-related issues.

It is important to notice here the context of development in Vietnam. First, with the development in the infrastructure, both physical and digital, one can expect the situation for entrepreneurship research, especially those related to technological utilization, can quickly take off. Second, universities and research institutions in Vietnam are still trying to catch up with the changes: new technological concepts like DOI, Data storage, preprints, open-access are slowly emerging among scholars in Vietnam (Vuong, 2019b). In medicine, Vietnam is gradually adopting artificial intelligence while battling the slow and outdated bureaucracy (Vuong et al., 2019). One can expect that once the academic community in Vietnam is fully integrated into the global community, they will be better equipped to produce more quantity and higher quality entrepreneurship studies. The question is how to speed up and move beyond the influences of these natural developments.

Starting with the research landscape provided in this study, higher education in policy-makers could incentivize research on under-studied topics, namely technology utilization, education and training, and gender/sex issues in entrepreneurship studies. Other areas of studies that should be more encouraged so that entrepreneurship research Vietnam can catch up with the trend of the world are the cognitive and theoretical aspects of entrepreneurship (Ferreira et al., 2019a; Meyer et al., 2014). Built upon well-established results highlighted in this review, future research should pay more attention to the determinants or the measures of success in the adoption of business practices aim to create a more innovative environment. In addition, there are also opportunities for studies on how to harness the cultural forces in entrepreneurial endeavors. Finally, although the Vietnamese government has used the term “an ecosystem of start-ups” regularly in Vietnamese media, Vietnamese researchers have not touched upon the idea of “entrepreneurial ecosystem,” which would promote and support entrepreneurial activities through incubation, mentoring, networking, etc. Evidently, with a clear plan to stimulate research on entrepreneurship based on an in-depth understanding of the existing research landscape, policy-makers can help researchers to be more engaged with the evolutions on entrepreneurship in Vietnam, while, maximizing the economic efficiency in the process (Vuong, 2018). Up-to-date and fresh insights by the research communities not only help businesses to make better decisions but also indirectly lay a foundation for a more sustainable future for society.

## Declarations

### Author contribution statement

H.M. Toan: Conceived and designed the experiments; Contributed reagents, materials, analysis tools or data; Wrote the paper.

Q.H. Vuong: Conceived and designed the experiments; Analyzed and interpreted the data; Contributed reagents, materials, analysis tools or data.

V.P. La: Conceived and designed the experiments; Performed the experiments; Contributed reagents, materials, analysis tools or data.

T.T. Vuong and T.H.K. Nguyen: Performed the experiments; Analyzed and interpreted the data; Wrote the paper.

H.M. Tung: Analyzed and interpreted the data; Contributed reagents, materials, analysis tools or data; Wrote the paper.

### Funding statement

This research is funded by Vietnam National Foundation for Science and Technology Development (NAFOSTED) under the National Research Grant No. 502.01-2018.19.

### Competing interest statement

The authors declare no conflict of interest.

### Additional information

The dataset is publicly available in OSF. DOI: 10.17605/OSF.IO/NJMSY. URL: https://osf.io/njmsy.
